# New Insights into Wnt–Lrp5/6–β-Catenin Signaling in Mechanotransduction

**DOI:** 10.3389/fendo.2014.00246

**Published:** 2015-01-20

**Authors:** Kyung Shin Kang, Alexander G. Robling

**Affiliations:** ^1^Department of Anatomy and Cell Biology, Indiana University School of Medicine, Indianapolis, IN, USA; ^2^Department of Biomedical Engineering, Indiana University–Purdue University at Indianapolis, Indianapolis, IN, USA; ^3^Richard L. Roudebush VA Medical Center, Indianapolis, IN, USA

**Keywords:** Wnt, Lrp5, Lrp6, Sost, mechanotransduction

## Abstract

Mechanical loading is essential to maintain normal bone metabolism and the balance between bone formation and resorption. The cellular mechanisms that control mechanotransduction are not fully defined, but several key pathways have been identified. We discuss the roles of several components of the Wnt signaling cascade, namely Lrp5, Lrp6, and β-catenin in mechanical loading-induced bone formation. Lrp5 is an important Wnt co-receptor for regulating bone mass and mechanotransduction, and appears to function principally by augmenting bone formation. Lrp6 also regulates bone mass but its action might involve resorption as well as formation. The role of Lrp6 in mechanotransduction is unclear. Studies addressing the role of β-catenin in bone metabolism and mechanotransduction highlight the uncertainties in downstream modulators of Lrp5 and Lrp6. Taken together, these data indicate that mechanical loading might affect bone regulation triggering the canonical Wnt signaling (and perhaps other pathways) not only via Lrp5 but also via Lrp6. Further work is needed to clarify the role of the Wnt signaling pathway in Lrp5 and/or Lrp6-mediated mechanotransduction, which could eventually lead to powerful therapeutic agents that might mimic the anabolic effects of mechanical stimulation.

## Introduction

Mechanical loading is essential to maintain normal bone metabolism and the balance between bone formation and resorption (1, 2). Disuse of bone or insufficient loading can cause reduced bone formation as well as increased bone turnover, which can be appreciated in astronauts that have spent significant time in space, among patients restricted to bed rest, and in people suffering from muscle paralysis (3). Conversely, exposure to frequent, high-impact loading results in active bone formation (4). A good example of this effect can be seen among competitive racquet sports players, in whom the upper limb bones of the dominant (playing) arm is generally more robust compared to the non-playing skeletal elements (5). These effects are consequences of Wolff’s law (6) and have been verified in numerous murine models of mechanotransduction, such as ulna- and tibia-loading models, the tail suspension model, and the botulinum toxin paralysis model (7–9). However, the precise physical and biological mechanisms that drive adaptation to disuse/overuse have not been completely elucidated.

**Figure 1:**
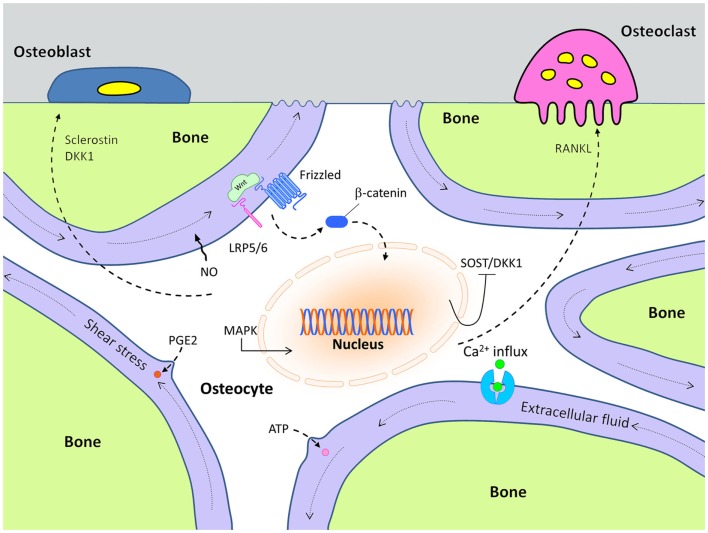
**Cellular mechanisms of action in mechanically stimulated osteocytes, which control osteoblast and osteoclast activity**.

Although the biological mechanisms that control mechanotransduction are far from certain, considerable progress has been made in identification of some of the key players in this process. For example, very early after a mechanical signal is received by bone cells, adenosine triphosphate (ATP) is released from stimulated cells and intracellular Ca^2+^ concentration increases within 1 min after loading (10–12). Subsequently, at least two other essential second messengers, prostaglandin E2 (PGE2) and nitric oxide (NO), are released from the mechanically stimulated cells (13–15) (Figure 1). Those events lead to the induction of MAP-kinase signaling (ERK 1/2) and C-fos expression (16, 17). Later, inhibition of Sost and Dkk1expression, along with increased expression of IGF-I and other bone matrix-related genes such as osteopontin and collagen have been documented (18, 19).

In this process, the Wnt pathway has been reported to play an important role in transmitting mechanical signaling in bone remodeling through both activating and inactivating several signaling cascades (20). The canonical Wnt pathway is initiated when released Wnt proteins bind to receptor complexes including a seven pass transmembrane co-receptor called Frizzled, and either low-density lipoprotein-related receptor-5 (Lrp5) or -6 (Lrp6). Formation of this receptor complex at the cell surface eventually leads to increased β-catenin-induced transcriptional activity via increased β-catenin levels in the cytoplasm.

Over the past decade, this Wnt/β-catenin signaling pathway has been identified as an essential component of mechanically induced signal transduction. Heterozygous deletion of β-catenin in osteocytes has been reported to significantly reduce loading-induced anabolic responses in bone (21). Wnt/β-catenin signaling in bone remodeling is proposed to be regulated by both Lrp5 and Lrp6 (22–25). Although Lrp5 and Lrp6 have common properties, such as sequence and structural similarity within this subfamily of the LDL receptor-related proteins (26–28), their molecular downstream events might be different in the context of bone remodeling. In this review, we discuss the roles of both Lrp5 and Lrp6 in mechanical loading-induced Wnt/β-catenin signaling pathways in the process of bone remodeling.

## LRP5

### Loss-of-function studies

Experiments performed on mice engineered with loss-of-function mutations in Lrp5 have revealed that Lrp5 is important to transfer the mechanical loading-induced signals in mechanosensory bone cells. Sawakami et al. investigated the crucial role of Lrp5 on skeletal mechanotransduction using mice with loss-of-function mutations (Lrp5-null) (25). Similar to the human patients with loss-of-function mutations in LRP5, the loss of Lrp5 signaling in mice yielded a significant decrease in bone-mineral density and bone strength. Mechanotransduction studies in these mice showed a reduced osteogenic response to mechanical loading, which was due to a reduction in load-induced synthesis of bone matrix, but not due to osteoblast recruitment and/or activation on the surfaces. Saxon et al. confirmed that Lrp5 has an important role in mechanotransduction using a different Lrp5^−/−^ mouse model and a different skeletal loading modality. Among the experiments they reported that where their control mice (Lrp5^+/+^) exhibited dose-responsiveness to loading, they observed a loss of responsiveness in the Lrp5 mutants (29).

Based on the positive effects of the mechanical loading on bone formation, we can surmise that loading might either increase expression of Wnt stimulatory molecules, reduce expression of inhibitory molecules, or both. One of the more notable inhibitors of bone formation is sclerostin, which is known to participate in the Wnt signaling pathway (Figure 1). Sclerostin is an Lrp5/Lrp6 antagonist that is encoded by the Sost gene. It is primarily expressed in the osteocyte population, and loss-of-function mutations in Sost (or in its regulatory elements) are known to cause sclerosing bone disorders such as sclerosteosis and Van Buchem disease (30–32). Sost transcript expression and sclerostin protein levels are dramatically decreased as a result of mechanical loading, especially in the high strain regions of the bone (8). Conversely, skeletal disuse causes an increase in Sost expression. The functional role of Sost regulation during mechanical loading was addressed by Tu et al., who showed that if the normal decrease in Sost expression that occurs during loading was prevented, the anabolic effects of loading were abolished (33). They accomplished this effect by loading engineered mice that harbored a transgene comprising an 8 kb Dmp1 promoter driving a human SOST cDNA. In these mice, the normal drop in endogenous Sost levels that occurs during loading was countered by an increase in human SOST during loading, owing to the load-sensitive Dmp1 promoter that regulated the hSOST cDNA.

### Gain-of-function studies

Other mutations in LRP5 have been identified in the human population that, rather than causing loss-of-function and very low bone mass, were found to cause abnormally high bone mass (HBM). Engineered mice have been generated to study the effect of these gain-of-function missense mutations in LRP5, including transgenic and knock-in models. Published reports from these mouse models demonstrate that the gain-of-function mutations in Lrp5 are associated with an HBM phenotype (24, 29). The mechanotransduction phenotype observed in the loss-of-function (Lrp5^−/−^) mice prompted several investigators to ask whether the HBM-causing mutations in Lrp5 also affect mechanotransduction, perhaps in the converse (beneficial) direction. To this end, Robinson et al. conducted ulnar loading experiments in mice harboring a human cDNA for LRP5 that included the G171V-causing nucleotide substitution (34). The cDNA was driven by the 2.3 kb Col1a1 promoter, which provided specificity of the HBM allele to mature osteoblasts and osteocytes. They showed that Wnt/β-catenin target gene expression was increased after loading and this effect was associated with increased cell responsiveness to mechanical loading. This result was confirmed by Saxon et al., who reported the same mouse model (2.3 kb Col1a1 – G171V) subjected to tibial loading produced an increased osteogenic response to mechanical loading (29). They also indicated that these HBM transgenic mice had increased resistance to bone loss associated with disuse compared to wild type (WT) controls.

The effect of HBM-causing mutations in Lrp5 on mechanotransduction were further probed by Niziolek et al., who used two novel HBM knock-in models to elucidate the role of Lrp5 in the loading response. Those investigators used a different approach than the G171V transgenic approach used by Robinson et al. (34) and Saxon et al. (29). Rather than overexpressing cDNAs for HBM-causing alleles only in osteoblastic cells, Niziolek et al. knocked in the G171V and A214V HBM-causing mutations into the endogenous loci. This strategy allowed for normal expression levels and tissue distribution, owing to the undisturbed promoter and regulatory elements. These mice are therefore a more orthologous model to two of the human HBM families (7). When axial tibia loading was applied for 3 days to mature male Lrp5 G171V and Lrp5 A214V knock-in mice and to their WT controls, fluorochrome-labeling results showed that this loading resulted in a significantly enhanced periosteal response in the A214V knock-in mice, whereas the G171V knock-in mice exhibited greater bone formation on the endocortical surface. This bone formation difference in two different HBM-inducing Lrp5 mutations indicated that these types of mutations can alter the mechanisms responsible for anabolic mechanotransduction, and also, that a portion of the HBM phenotype observed in human patients carrying gain-of-function mutations in LRP5 might be at least partially due to enhanced mechanoresponsiveness in their skeletons.

## LRP6

Lrp6 is similar in sequence and structure to Lrp5, and these two receptors have been proposed to function largely in the same contexts and signaling pathways. However, there are some important differences between the two receptors. The most obvious difference is that whereas Lrp5^−/−^ mice are viable and fertile (albeit with low bone mass), Lrp6^−/−^ mice exhibit an embryonic lethal phenotype. Those observations suggest that either (1) the timing, location, and/or level of embryonic expression is different between Lrp5 and Lrp6, (2) the ligands, inhibitors, and/or downstream cascades differ between these two receptors, or (3) some combination of those two possibilities. For example, Lrp6 appears to be crucially important for bone’s anabolic response to intermittent parathyroid hormone (PTH) treatment, whereas Lrp5 appears to be uninvolved. Li et al. showed that deletion of a floxed Lrp6 allele in osteoblasts, using osteocalcin-driven Cre recombinase, disrupts the bone anabolic activity of PTH by reducing number of osteoblasts (23). Wan et al. showed direct interaction between Lrp6 and the PTH 1 receptor using fluorescence resonance energy transfer (35). While Lrp6 appears to be important for PTH signaling, at least two published reports indicate that Lrp5 is not essential for transducing PTH signaling (25, 36). These data suggested that Lrp5 and Lrp6 might participate selectively and differently in Wnt and/or other hormonal signaling. On the other hand, while Lrp5 is crucial for mechanotransduction, there is to date no clear evidence indicating that Lrp6 participates in mechanical loading-induced activation of Wnt signaling pathway.

Although both Lrp5 and Lrp6 are required to develop normal postnatal bone, available evidence indicates that their roles in this process might differ. Lrp6 has been reported to play a role in bone resorption and formation, whereas Lrp5 appears to affect bone formation but not bone resorption (22–25). These observations come from a number of studies that have looked at cell-specific effects of Lrp5 and Lrp6 in bone. For example, Kubota et al. reported the discovery of a naturally occurring mutation in Lrp6, which conferred hypomorphic properties to the receptor (referred to as the “*rs*” mutation). In a careful analysis of these mice, they found that canonical Wnt signaling was severely impaired in cells harvested from the rs/rs mice compared to WT mice (22). As expected, the rs/rs mice displayed low bone mass *in vivo*, but the phenotype was primarily due to increased bone resorption, with no detectable change in bone formation. This result stands in stark contrast to that reported for Lrp5^−/−^ mice, in which bone resorption is normal but bone formation is reduced considerably. While both Lrp5 and Lrp6 can regulate Wnt signaling, the receptors might be active in osteoblasts over different time windows. A recent report indicates that Lrp6 might affect early osteogenic differentiation, whereas Lrp5 seems to affect late osteogenic differentiation (24). It should also be noted that the resorption/formation phenotype observed in the Lrp6 hypomorphic mice was not confirmed in an osteoblast-specific deletion model (Ocn-Cre with Lrp5^flox/flox^ mice); in fact, the opposite mechanism was reported, i.e., reduced bone formation and no change in resorption (23, 24). Li et al. observed that Lrp6 KO mice showed little change in the osteoclast number but reduced the osteoblast number compared with the WT littermates (23).

Another remarkable point of difference between Lrp5 and Lrp6 function is that the bone compartment affected might be different, although both Lrp5 and Lrp6 can both influence cortical bone remodeling. Sawakami et al. showed that the cross-sectional area of ulnas from Lrp5-deficient mice was reduced compared to WT mice (25), whereas Lrp6 appeared to be involved preferentially in trabecular bone development (22). This suggests that the developmental role of Lrp5 and Lrp6 might be different across bone surfaces. Other examples of compartment-specific effects have been reported for the Wnt cascade. For example, Liu et al. observed a surface-specific influence of Wnt signaling on bone metabolism. They found that Wnt16 deletion decreased cortical bone thickness and increased cortical bone porosity, while very minor changes were observed in trabecular bone in mice lacking Wnt16 (37).

## β-Catenin

A major canonical downstream target of both Lrp5 and Lrp6 is β-catenin. Inactivation of β-catenin in osteoblasts (e.g., 2.3 kb Col1a1-Cre crossed to β-catenin^flox/flox^) causes osteopenia by affecting bone resorption rather than bone formation (38). The resorption phenotype in these mice has been replicated using Dmp1-Cre, a Cre driver that is active later in the osteoblast differentiation pathway, i.e., late-stage osteoblasts and osteocytes. Osteocyte-specific β-catenin-deficient mice showed a low bone mass phenotype via increased osteoclast number and activity, while osteoblastic function was normal (39). Mechanistically, downregulated osteoprotegerin (OPG) expression was implicated in osteoclastic resorptive activity effect of those mice. Because β-catenin is downstream of Lrp5/6, this result is consistent with the Kubota et al. report on the Lrp6 hypomorphic mouse, but is inconsistent with the reports on Lrp5^−/−^ and osteoblastic Lrp6^−/−^ mice. It is interesting to note that the β-catenin resulted in a more severe bone phenotype than lack of either Lrp5 or Lrp6, indicating that there might be a combined (or synergistic) effect of Lrp5 and Lrp6 on bone regulation. Holmen et al. showed this possibility by comparing mice lacking various combinations of Lrp5 and Lrp6 (40). Additionally, β-catenin receives input from other proteins (e.g., mTOR, Pi3K), so it might be unreasonable to expect that β-catenin deletion would phenocopy the deletion of Lrp5/6. Regarding the role of β-catenin in mechanotransduction, it was recently shown that both copies of β-catenin are required in osteocytes and/or late-stage osteoblasts for mechanotransduction to occur; mice haploinsufficient for β-catenin in those cell populations showed no measureable response to mechanical stimulation *in vivo* (21).

Based on the various evidences regarding the Lrp5 function on bone formation and the Lrp6 role on bone regulation via bone resorption, we can hypothesize that the mechanical loading might affect bone regulation triggering the canonical Wnt signaling not only via Lrp5 (bone formation) but also via Lrp6 (perhaps both bone resorption and formation). In normal conditions, Wnt signaling pathways probably are influenced by both Lrp5 and Lrp6 at the same time, but in different ways and their roles might be synergistic. Further investigation on the role of Lrp6 in mechanical loading-induced signaling pathways and the synergistic effect of Lrp5 and Lrp6 could show more clearly the mechanism of Wnt signaling, via both Lrp5 and Lrp6, in mechanotransduction.

## Conclusion

Mechanical loading is a powerful modulator of bone modeling and remodeling. The exact cellular and molecular mechanisms by which this process occurs are still unclear. Substantial evidence indicates that the Wnt signaling pathway participates in the transduction of mechanical signals at the cell surface and ultimately leads to the regulation of bone metabolism. Lrp5 is intricately involved in bone cell mechanotransduction, but there is no indication at this time whether Lrp6 has any role in this process. Further studies are needed to clarify the role of the Wnt signaling pathway in Lrp5 and/or Lrp6-mediated mechanotransduction, which could eventually lead to powerful therapeutic agents that might mimic the anabolic effects of mechanical stimulation.

## Conflict of Interest Statement

The authors declare that the research was conducted in the absence of any commercial or financial relationships that could be construed as a potential conflict of interest.
